# Insights on Natural Membrane Characterization for the Rational Design of Biomimetic Drug Delivery Systems

**DOI:** 10.3390/pharmaceutics17070841

**Published:** 2025-06-27

**Authors:** Daniela Donghia, Sara Baldassari, Giuliana Drava, Giorgia Ailuno, Gabriele Caviglioli

**Affiliations:** Department of Pharmacy, University of Genova, 16148 Genova, Italy; daniela.donghia@edu.unige.it (D.D.); sara.baldassari@unige.it (S.B.); giuliana.drava@unige.it (G.D.); gabriele.caviglioli@unige.it (G.C.)

**Keywords:** cell membrane, extracellular vesicles, exosomes, lipidomics, proteomics, atomic force microscopy, biomimetic vesicles, bioinspired nanosystems, hybrid liposomes

## Abstract

Cell membranes are vital for living organisms and serve as a dynamic barrier between the internal and external environments. They are composed of a complex lipid bilayer embedded with proteins, allowing them to perform multiple functions like maintaining the cell structure, regulating which substances enter or leave the cell, and intercellular communication. Cellular functions are inherently linked to their membrane properties, and the heterogeneous nature of cell membranes makes the study of their physico-chemical properties extremely challenging. This review is intended to provide a comprehensive overview of the composition and physical features of the cell membrane, by focusing on the lipid and protein composition, and on the physical properties (like membrane stiffness or fluidity), highlighting how these characteristics influence cell functions. An insight into the similarities and differences from the membranes of extracellular vesicles (naturally secreted by almost all cell types) is also provided. The understanding of the physico-chemical properties of cell membranes might find application in different therapeutic fields, like disease diagnosis and development of novel drug delivery systems. Therefore, an overview of the literature works describing the rational design of biomimetic drug delivery systems is presented, focusing on the choice of lipid components, frequently inspired by the study of the composition of naturally secreted exosomes, and on the physical characterization of the systems. In the future, in-depth study of biologic vesicles might lead to the development of promising formulation for drug delivery, possibly enhancing the therapeutic outcomes of many pathologies, like cancer.

## 1. Introduction

Cell membranes are fundamental components of all living organisms. They serve as a dynamic barrier that separates the interior of the cells from their external environment. Thanks to their intricate structure, cell membranes can complete pivotal functions such as maintaining cellular integrity and facilitating vital biological processes [1]. The cellular membrane plays crucial roles in protecting the cell, facilitating intercellular communication, regulating the internal and external cellular environments, and managing metabolic processes [2].

Cell membrane architecture primarily consists of a lipid bilayer with proteins embedded within it. This structure grants membrane selective permeability, facilitates communication, and supports the transport of substances both into and out of the cells [3]. The fluid mosaic model, proposed by Singer and Nicolson in 1972 [4], remains, even nowadays, a cornerstone in visualizing the cellular membrane, to understand its dynamics and study how lipids and proteins interact within this bilayer.

Studying the physico-chemical characteristics of cell membranes is crucial, due to their profound impact on cellular function and in view of potential applications in biotechnology and medicine. For example, Tan et al. underline that the prediction of the biological fate of biopolymer-liposome hybrid carriers would require a thorough understanding of its influencing factors, two of the main ones being physico-chemical properties of the carriers and the way they interact with biological systems [5]; while Rodriguez et al. stress how the identification of individual membrane characteristics enables researchers to formulate better biomimetic platforms for various applications, one of them being advanced drug delivery [6]. Indeed, the membrane components, in particular the proteins, may delay macrophage clearance, increasing circulation time, and augment the uptake by the target cells, providing an overall improvement in drug delivery with respect to platforms made of purely synthetic components. However, understanding the complex dynamics of cell membranes presents several challenges, the most significant of which is their heterogeneous nature. The membranes are composed of diverse constituents, and the tools suitable to investigate protein dynamics within their native lipid environments are scarce [7]. The heterogeneous nature of membranes, with their diverse lipid and protein constituents, poses a substantial obstacle to advancing our knowledge in the field. This topic becomes even more complex when considering the membrane composition of the plethora of vesicles produced by the cells, which might not correspond exactly to the composition of the parent cell membranes.

This literature review aims to summarize current knowledge of the physical and chemical properties of the membranes of cells and extracellular vesicles (EVs), highlighting the similarities and the differences occurring between them; moreover, since this knowledge might be successfully employed to the rational design of bioinspired systems for therapeutic applications, this review also aims to discuss how this information has been applied to the development of biomimetic drug delivery systems so far.

## 2. Cell Membrane Characteristics

### 2.1. Lipid Composition

Lipids have various functions in cells as structural components of the membrane [8], as energy and heat sources [9], as signaling molecules [10], as protein recruitment platforms [11], and as substrates for post-translational protein-lipid modification [12]. The lipidic composition of the membranes may influence their fluidity and even their selectivity in internalization processes. For example, motile lymphocytes are characterized by low cholesterol (chol) level and high content of unsaturated lipids, while non-motile tissues, for example the ones that synthesize chol, present a high level of chol [13]. The lipid bilayer of the membrane provides a functional barrier between the cell and its surrounding environment. The physical–chemical properties of lipids are mainly influenced by their chemical structures, therefore it is important to understand how lipid structures and their local composition are translated into functions [9]. Dysregulation of lipids of the membranes is shown to be connected to a significant alteration of cell function and morphology, leading to different kinds of diseases [14]. Understanding these changes is essential for developing novel therapeutic strategies. Indeed, a relevant number of diseases seems to be associated with mutation in enzymes involved in lipid metabolism, demonstrating how lipids present multiple functions in physiology [15].

The lipid fraction of the plasmalemma could be divided into phosphatidic acid derivatives, glycolipids, sphingolipids and chol [16]. Table 1 summarizes the main lipid components of membranes, their distribution between the leaflets, and their biological functions.

The phosphatidic acid, constituted of a diacylglycerol backbone and a phosphate group accumulating on the cytoplasmic side [17], usually does not exceed 1 mol%, but still plays a significant role due to its negative charge. It is regulated by diacylglycerol kinase (DGK) and phospholipase D; DGK activity is hormonally regulated and can be activated by vascular endothelial growth factor (VEGF) and pathogens. Elevated phosphatidic acid levels activate kinases involved in cancer cell metabolism and proliferation, more specifically implicated in intracellular stress signaling pathways. Moreover, the elevated level of phosphatidic acid enhances hypoxia-inducible factor 1-alpha, promoting angiogenesis [17,18].

The phosphatidylinositol (PI) family is a diverse group of lipids with varying fatty acid compositions and phosphate groups. PI levels fluctuate in different cell types, between 5 and 12 mol%, with specific roles in membrane trafficking and proliferation. PI trisphosphate accounts for numerous cellular signaling pathways activation, contributing to cell growth and survival regulation [19].

Phosphatidylglycerol (PG) is present to a lesser extent in cell membranes, representing only about 1 mol% of the membrane phospholipids, but it is a crucial component of the plasma membrane, particularly during viral infections; abnormally high PG levels may correlate with increased viral replication and cancer development [18,20].

Phosphatidylserine (PS) is a negatively charged lipid, primarily found in the inner membrane layer (around 6 mol%) [18]. Its negative charge contributes structurally to the membrane curvature and fluidity, and the ability of proteins with poly-cationic domains to dock the membrane is helped by its electrostatic charge [21]. Its degradation is linked to the biosynthesis of phosphatidylethanolamine, which occurs mainly in the plasma membrane. PS plays a role in apoptosis signaling and can protect cancer cells from immune detection. This happens when PS passes to the outer layer of the cell membrane due to the activity of an enzymatic protein called calcium ion-dependent flippase [22]. Indeed, when PS is normally concentrated in the inner leaflet of the membrane, it electrostatically interacts with positively charged domains of cytosolic and signaling proteins; when it is externalized, these proteins are released, while coagulation factors and proteins involved in immunoregulation are bound and their biochemical pathways are activated [23]. Recent findings suggest that, in cancer cells, PS can also be exposed during cellular oxidative stress, indicating a shift from apoptotic to necroptotic cell death. This exposure exerts a dual function: besides marking the cell for immune recognition, it also helps cancer cells evading immune detection by masking their presence from natural killer cells and other immune cells. Due to this ability, PS can be considered as a tumor marker [18,24]. On the basis of this consideration, a few therapeutic agents have been developed that specifically target PS: as an example, bavituximab binds PS when recognizing a complex formed by the phospholipid and two cross-linked molecules of the PS-binding protein beta 2 glycoprotein-1 [25]; due to its large size, the antibody molecule can bind the complex only when PS is exposed on the membrane outer surface, conferring it very high specificity against those cells where translocation has occurred, namely tumor cells [25]. Moreover, PS favors the fusion of EVs membrane with target cell membranes [26].

Phosphatidylethanolamine (PE) is a prevalent membrane phospholipid, around 15 to 25 mol%, with a higher concentration in the cytosolic leaflet. In the membrane PE, normally being in a lamellar phase, can assume a hexagonal II phase that might enhance the fusion of lipid bilayers with lysosomal membranes [26]. Its distribution may reverse in cancer cells, affecting membrane protein folding and function. PE is involved in many roles: first, it operates as a lipid chaperone, facilitating membrane protein folding, hence its deficiency can lead to unfolded proteins that drive a metabolic shift toward a cancerous state; it also modulates membrane protein activity in signaling pathways [18], supports autophagy, and regulates protein interactions, all of which play a role in cancer progression [18,20,27]. This might apparently be due to the uncharged nature of this phospholipid, which might shield negatively charged aminoacid residues in proteins, increasing the topogenic effect of positively charged residues, finally affecting the position and function of membrane proteins [28].

Phosphatidylcholine (PC) is a neutral lipid found in both ester and ether forms. It is the most abundant compound of the lipid bilayer (around 40–50 mol%) [29] and is primarily present in the outer leaflet. Altered PC metabolism is linked to various cancers, driven by specific enzyme activities such as choline kinase alpha and phospholipases C and D. Variations in PC levels can affect cell proliferation and energy metabolism in cancer cells [18].

Sphingolipids are composed of a molecule of sphingosine linked to fatty acids or phosphate derivatives (ceramide and sphingomyelin (SM), respectively) [30]. Even if they are a minor class of phospholipids, sphingolipids are involved in numerous functions relevant to cell development and biology, such as molecular sorting, cell–cell interaction, intracellular transport and signaling [31]. Due to their physico-chemical characteristics, sphingolipids interact more favorably with each other and with sterols than with other lipids [32]. While the chemical differences among glycerophospholipids derive from the combination of the two fatty acids, which can be saturated or unsaturated, and from the different head groups [33], for sphingolipids the chemical differences arise from the variation in the head group, in the N-acyl chain and in the length and type of the sphingoid base. In sphingolipids, the N-acyl chain tends to be more saturated and longer than the acyl chain of glycerophospholipids, making membranes with a higher content of sphingolipids thicker and less fluid [34].

The head group of phospholipids acts as a “code” that mediates the biological function of the lipid through its interaction with proteins [35]. The size of the head group and the hydrophobic tails of lipids can affect the shape of the lipids and, consequently, the membrane curvature [36], and also influence membrane fluidity. For example, due to the different size of the head group, PC, with its large head group, can increase membrane fluidity, since choline occupies an area that is similar to the one of the fatty acyl chains; conversely, PE, with its slightly smaller head group, reduces membrane fluidity, since ethanolamine occupies a larger area than the fatty acyl chains. PC and PE also have an effect on hydration: PC is more hydrated than PE, resulting in a looser packing of adjacent hydrophobic lipids; PE can form hydrogen bonds with the anionic phosphate-oxygen of the adjacent phospholipid, restricting its movements and leading to more rigid membrane structures. PC and SM have the same choline head group, but SM results in being less hydrated, and hence more tightly packed [37]. Furthermore, the head group has a marked influence on the surface charge of the cell membrane. Usually, the bilayer phospholipid membranes exhibit a negative surface charge due to ions that are trapped in potential wells formed by the dipole heads of the phospholipids. This negative charge influences the binding energy of ions to the membrane [38]. Some studies discuss the implications of surface charge on pore dynamics, particularly in relation to the transport of large molecules and ions, such as water and hydrogen peroxide. The modification of surface charge due to interactions with external factors represents a critical aspect of pore formation and transport properties [39]. Moreover, the cell surface charge influences the effect of drugs, as those with opposite charges feature enhanced binding to the membranes and intracellular delivery [40].

Phospholipid distribution in the outer and inner leaflet of plasma membrane is not symmetrical. The asymmetric distribution of phospholipids, critically contributing to the biophysical properties of the cell membrane, influences factors such as membrane fluidity, curvature, and the distribution of transmembrane potential. The importance of membrane asymmetry extends far beyond structural stabilization; functionally, the intracellular localization of anionic phospholipids like PS underlies the activation and regulation of numerous membrane-bound enzymes involved in signal transduction and ionic homeostasis. Moreover, it has been demonstrated that membrane asymmetry helps prevent problems such as blood coagulation and aids in properly orienting membrane proteins, but our understanding of the reasons for maintaining membrane asymmetry remains rather circumstantial. From a mechanistic perspective, maintaining an asymmetric distribution of lipids consumes substantial amounts of free energy, which is expended through the activity of integral membrane proteins involved in lipid translocation, including flippases, floppases, and scramblases [41], able to oppose spontaneous lipid flip-flop that would otherwise lead to a collapse of the membrane functional polarity [42]. Which processes are involved in maintaining this homeostatic asymmetry is a challenging question, which has found tentative responses in the study of relatively simple species (such as specific kinases and transcription factors in *Saccharomyces cerevisiae* [43]), responses that might not apply to higher species. Also, the factors that might alter physiologic asymmetry, such as an increase in calcium levels or chol depletion, are certainly numerous but hardly defined [44].

Even if a deeper understanding in lipid asymmetry might provide key insights into a plethora of physiological processes related to plasma membranes, issues regarding the challenging construction of asymmetric membranes with precisely defined lipid compositions has hampered the research in this field [45]. In this context, an interesting study was carried out by London [46], who proposed a model of asymmetrical lipid membrane, better mimicking real biological membranes. To prepare the asymmetric lipid vesicles, London exploited the lipid exchange method: an excess of vesicles composed of lipids that will constitute the outer leaflet is mixed with a second set of vesicles composed of the lipids that will be part of the inner leaflet, in the presence of cyclodextrins, serving as water-soluble agents for lipid exchange.

The availability of models mimicking natural membrane asymmetry is crucial not only for understanding biological mechanisms and alterations being responsible of diseases, but also for developing drugs that are efficiently internalized within cells. In fact, traditional symmetric models employed to study permeation may show critical weaknesses, and must be used with caution with the aim of predicting permeation of metabolites and active substance through natural membranes [47]. Membrane asymmetry should also be pursued in the development of effective drug nanocarriers, including liposomes and biomimetic vesicles, to obtain particles having a closer resemblance to natural vesicles. Different methods, including the aforementioned application of cyclodextrin-driven exchange or microfluidics, have been successfully employed to obtain nanovesicles with inner leaflet differing from the outer one, having better drug loading ability, stability and targeting capability [48].

Besides phospholipids, important cell membrane components are sterols. In eukaryotic cells, sterols are the second most abundant group of lipids after phospholipids, with chol being the principal sterol in the mammals, playing a fundamental role in regulating membrane permeability [49]. Chol, whose content in cell membranes usually varies between 20 and 50 mol%, is the final product of a sterol biosynthetic pathway that involves more than 20 enzymes [50]. Filling the gaps between phospholipids in the membrane bilayer, chol significantly affects membrane parameters like stiffness, thickness and thermosensitivity (phase transition temperature); in fact, the phospholipid/chol ratio is a parameter usually employed to assess membrane stiffness. Chol orders the fluid phase lipids in the membrane, producing a condensing effect on this lipid order leading to an increase in the bilayer thickness and a decrease in its permeability. This effect, however, depends on the type of lipids, as lipids with multiple unsaturations apparently inhibit its condensing effect [51]. An example of the condensing effect of chol is shown by Gumì-Audenis et al. [52]: they analyzed the breakthrough force (Fb), obtained by Atomic Force Microscopy (AFM), of bilayers composed by DOPC (1,2-dioleoyl-*sn*-glycero-3-PC) or DLPC (1,2-dilauroyl-*sn*-glycero-3-PC) and observed an increase in Fb values, indicating an enhanced order and packing of the membrane, when the amount of chol increased up to 50 mol%.

Within the phospholipid bilayer, the distribution of chol between lipid monolayers is often asymmetric and generally depends on the distribution of phospholipids with anionic head groups and saturated chains. Indeed, chol exhibits a different affinity for different phospholipids: the strongest interaction occurs with sphingolipids and, consequently, this may favor its accumulation in the outer leaflet; on the other hand, PE and PS may also condition chol accumulation in the inner lipid monolayer [53]. According to theoretical calculations, approximately 60% of membrane chol is located in the inner monolayer [54]. The distribution of lipids and chol in the lipid monolayer is organized into tightly and thicker packed membrane domains termed “rafts”, rich in chol and SM [32,55]. These lipid-ordered domains, recruiting and laterally segregating specific proteins, act as sorting platforms for protein export in selective transport carriers [56]. These rafts are fluctuating nanoscale assemblies of lipids and proteins that play important biological roles in membrane signaling and trafficking [57]. They also contribute to neoplastic or tumor growth and invasiveness [58]. Zhuang et al., for example, verified the role of chol in prostate cancer progression using mice xenograft models [59]. First, they used simvastatin to determine whether the inhibition of endogenous chol biosynthesis in prostate cancer cells could alter cell survival and signal transduction through the Akt serine-threonine kinase and its downstream effectors; simvastatin significantly decreased the chol content of lipid rafts, with consequent inhibition of Akt signaling and stimulation of apoptosis. The correlation was confirmed as the results were reversed after chol replenishment. They also observed that chol-depleting drugs did not significantly induce apoptotic effects in the untransformed cells as they did in androgen-sensitive human prostate adenocarcinoma cells, suggesting that certain prostate cancers might be susceptible to chol-targeted therapy, while normal cells seem to be more resilient to changes in chol levels. To further confirm the effects of increased/decreased chol levels, they fed mice bearing androgen-sensitive prostate cancer cell-derived xenografts with a high chol diet, obtaining a raft-dependent increase in Akt phosphorylation that promoted tumor growth and reduced apoptosis. Instead, the treatment of mice with simvastatin disrupted the raft domains in prostate cancer cells, reducing phosphorylation of Akt and inducing apoptosis.

In another study, the effect of chol concentration was evaluated on giant plasma membrane vesicles (GPMVs) secreted by different types of cells after chemical induction with dithiothreitol and paraformaldehyde or *N*-ethylmaleimide. Chol concentration in the membrane was altered by incubating the cells with methyl-β-cyclodextrin before GPMV isolation, in order to exploit the cyclodextrin for chol exchange. About 60% of the cellular chol was extracted and the GPMVs isolated from chol-depleted cells resulted to be softer than the ones isolated from untreated cells when analyzed by AFM [60]. These results corroborate the concept that a high percentage of chol in the membrane produces tighter rafts, generating a more rigid structure and reducing the bending ability of the membrane.

Lipid composition and organization can vary significantly across different cell types and conditions. For instance, neural cell membranes are particularly enriched in chol and sphingolipids, compared to other cell types [61,62,63].

Various studies highlight that the lipid composition plays a crucial role in several pathologies, especially when a mutation affects specialized pathways of cell membranes inducing genetic diseases. An example is described by Nakahara et al. [64], where the identification of enzymes involved in lipid metabolism uncovered the genetic disease Sjögren–Larsson syndrome. They demonstrated that the Sjögren–Larsson syndrome causative mammalian gene *ALDH3A2* is responsible for the conversion of the sphingosine 1-phosphate degradation product hexadecenal to hexadecenoic acid. The absence of *ALDH3A2* causes abnormal metabolism of hexadecenal to ether-linked glycerolipids.

Besides the mutation of single lipids, the redistribution of the number of components can disrupt the homeostasis of the membrane; for example, high levels of ceramides are found in diseases such as type-2 diabetes [62], cancer, Alzheimer’s disease, and cystic fibrosis [34,65].

In this respect, Escribá et al. [66] underline how many functions occur in and/or around membranes, suggesting that changes in the membrane composition and structure could be relevant to the proper functioning of the cells. Modifications in the types and abundance of lipids lead to alterations in the propagation of cellular messages, which can be associated with pathological states. This study also introduces the concept of membrane lipid therapy, which involves the pharmacological regulation of membrane lipid composition and structure for the treatment of diseases. The molecules developed to exploit this therapeutic approach target the membrane lipid boundary of cells and/or internal organelles, where the cellular functions occur. The key to this therapy is the identification of the factors regulating membrane lipid structures, and their roles in cell signaling and development in pathological conditions.

### 2.2. Protein Composition

Another chemical characteristic to take into consideration when studying cell membrane composition is the presence of proteins. Proteins contribute to preserving the structural organization of the membranes and are involved in the regulation of the flow of ions and molecules [3]. The integration of a protein in the lipid bilayer allows it to interact intimately with both the hydrophobic environment provided by lipids and the aqueous cellular environment, which is essential for maintaining the dynamic equilibrium of the cell. A primary function of membrane proteins is the facilitation of ion and solute transport across the otherwise impermeable phospholipid bilayer. They form channels and transporters that regulate the movements of ions, metabolites and signaling molecules, thereby establishing and modulating the electrochemical gradients critical for cellular processes such as energy production and signal transmission. In addition to transport, many membrane proteins serve as receptors for extracellular signals; for example, G protein-coupled receptors detect extracellular molecules and initiate intracellular signaling cascades that regulate a multitude of cellular responses, including metabolism, growth, and differentiation. Furthermore, membrane proteins play a pivotal role in maintaining the structural integrity and dynamic organization of the lipid bilayer itself. They interact with lipids to form distinct microdomains, which not only support the spatial organization of proteins, but also contribute to the mechanical properties of the membrane [67].

### 2.3. Lipid–Protein Interaction

Lipid–protein interactions have a fundamental role in many biological processes that are vital for the cells [68]. Various classes of proteins interact with the membrane, these interactions are governed by multiple parameters, including stereospecificity, hydrophobicity, and electrostatics. This complexity arises from the extended structure of amphipathic helices, which allows them to interact with many lipids simultaneously, as opposed to well-folded domains that may interact with a single lipid [69]. These interactions between lipids and proteins in a cell membrane are fundamental, since folding, and structure and function of membrane proteins are influenced by their lipid environment [70]. For example, the lipid composition, influencing deformability and the intrinsic curvature of the membrane, might affect the recruitment of BAR-domain-containing proteins, which are curvature-sensing proteins. Indeed, it has been observed that chol depletion causes an increase in PS density, inducing plasma membrane curvature, that facilitates the recruitment of some BAR-domain-containing proteins, like endophilin. This lipid–protein interplay is critical for events such as clathrin-mediated endocytosis, where membrane shape transitions are required to generate vesicles [71]. Lipid composition can affect transmembrane protein localization and conformation, as in the case of ion channels [72], or can exert lateral pressure on the protein, affecting its conformation [73]. In this context, Das and Eliezer [74] used different biomimetic lipid membranes as models to study protein–lipid interactions through biophysical methods. They studied various interactions, for example, the one between α-synuclein (α-syn, a small soluble protein that can aggregate and form fibrils that accumulate in the neurons, causing neurodegenerative pathologies) and biomimetic lipid membranes for its implication in Parkinson’s disease. Their study shows that α-syn binding affinity for the biomimetic membranes is directly correlated to the amount of negatively charged phospholipids. Another example of evaluation of lipid–protein interactions is the case of amyloid β peptide with large unilamellar vesicles composed of DOPC, revealing the role of the zwitterionic membrane lipid in increasing the fibril growth [75].

### 2.4. Physical Characteristics

From the lipid and protein composition and distribution we can derive some important physical features characterizing cells. Among the physical properties, mechanical properties, like rigidity, are often investigated. Cell mechanical properties depend first on cytoskeleton structure and composition; on the other hand, membrane composition, specially its content in chol, may influence cortical cell stiffness. The study of the mechanical properties of cells is a rapidly evolving interdisciplinary field that seeks to elucidate how mechanical forces regulate cell function, physiology, and disease conditions, such as growth, division, differentiation, proliferation, migration, and adhesion [76]. Some studies showed that variation in the mechanical properties of the cell can lead to the emergence and development of diseases. For example, elevated membrane stiffness is observed in different types of cancer or fibrotic diseases. This increased stiffness causes extracellular matrix remodeling, which leads to abnormal mechanical feedback, exacerbating disease progression. Conversely, abnormal softening might make cells more susceptible to mechanical deformations triggering pathological responses, such as abnormal cell migration or improper cell signaling [77]. Analyzing the mechanical properties of single cells might allow to detect abnormal cells, enabling early disease diagnosis and assisting in drug screening [78].

Different techniques have been employed to study the mechanical properties of cells.

#### 2.4.1. Atomic Force Microscopy (AFM)

One of the most widely used techniques to evaluate the mechanical properties of living cells is AFM. This microscopy technique leverages an indentation process, relying on the fluctuations of a contacting mechanical probe to produce details on the morphology and on the elasticity of an analyzed sample. Briefly, a sharp tip (Rc = 5–15 nm), attached to a cantilever, approaches the surface of the object thanks to the attractive forces between the surface and the tip. A deflection of the tip is induced by increasingly repulsive forces, as the cantilever is brought in contact with the sample surface [79]. A position-sensitive photo diode (PSPD) tracks the different directions of a reflected laser beam, incidental on the cantilever, thus detecting the deflections of the cantilever. Therefore, when an AFM tip passes over a region of interest, scanning a substrate surface and encountering different objects, the cantilever is deflected and the PSPD records the changes in direction of the reflected beam. The collected data on the forces that the sample imposes on the tip can be used to form an image of the topography of the sample surface or to measure its elasticity/stiffness, with the help of various theories and models [78,80].

For example, Wang et al. [78] took into consideration different models of cell membrane. Their purpose was to evaluate the pros and cons of each model, and to try to understand which one could best describe the mechanical properties of the cells they were analyzing. Even if none of the studied static models were chosen, they developed an interesting dynamic model based on the structure of a general Maxwell system to describe the cell deformation dynamics under constant indentation depth during the stress–relaxation phase, taking into account elasticity and viscosity as parameters that can be used to recognize the cell type.

The surface tension of the membrane has not been really taken into consideration until recent years, when the latest studies have shown its important role in cell mechanical behavior [81,82]. Cell membrane surface tension participates in regulating the transport through the membrane and influences cell local curvature, migration and polarity [83,84]. Leo et al. [85] studied the mechanical differences in red blood cells obtained from patients affected with cirrhosis and spur cell anemia before and after liver transplantation. They observed a shape transition associated with altered cell viscosity, both restored after transplantation, when mechanical measurements were conducted via AFM. The authors observed that the sigmoidal trends in the elastic modulus versus indentation speed curve shifted toward lower elastic modulus values, aligning more closely with those observed in the healthy control group.

Some differences have been highlighted in the mechanical properties of cancer and normal cells; apparently, cancer cells are less stiff than healthy ones. For example, Ren et al. [76] tested four breast cell lines (non-cancer and cancer cells, both human and murine) and three human urothelial carcinoma cell lines, characterized by different grade of malignancy; they analyzed the cytoskeleton network modulus Ec, the cytoplasmic viscosity ηc, the cytoplasmic diffusion coefficient Dc, and the membrane tension γ. In all cases, the authors observed that moving from the non-cancer cells to the corresponding cancer cells with higher malignancy, Ec, ηc and γ decreased, while Dc increased. This study corroborates the importance of mechanical studies in identifying modifications in the cells, possibly anticipating the diagnosis of diseases.

Similar findings are reported in the study conducted by Lekka et al. [86] to evaluate the differences in Young’s modulus value in cell lines of pancreatic and breast cancer with a growing stage of cancer progression. They demonstrated that the heterogeneity of cell structure plays a crucial role. Indeed, they observed that the more the stage of cancer progression was advanced, the more Young’s modulus decreased; conversely, normal cells tended to have a lower ability to deform, having a higher Young’s modulus value. This study shows that the heterogeneous nature of cell structure is a critical determinant in the measurement of cellular mechanics, that influences stiffness values and might be exploited in the detection of pathological variations.

#### 2.4.2. Scanning Electron Microscopy (SEM) and Transmission Electron Microscopy (TEM)

Besides AFM, other microscopy techniques, like scanning electron microscopy (SEM) and transmission electron microscopy (TEM), have been used to characterize the cell membranes from a physical point of view. In SEM, an electron beam with acceleration voltages (up to 30 kV) is focused on the specimen, causing electron emissions from the specimen. The emitted electrons are collected by the detectors; a secondary electron (SE) detector captures secondary electrons, containing information about the surface topography and composition of the sample; and a back-scatter electron (BSE) detector provides information about the elemental composition of the sample [87]. Moreover, the BSE detector enables the investigation of protein localization by using gold-conjugated secondary antibodies. Interestingly, Begemann et al. [88] proposed a novel SEM protocol, exploiting a combination of SE/BSE detection and gold-conjugated secondary antibodies to investigate the correlation between membrane ultrastructure and localization of proteins of interest (like, for example, curvature-sensitive proteins).

Compared to SEM, TEM can reach higher magnification, making it more suitable for measurements in the nanometer scale. Also, in TEM, an accelerated electron beam is focused on the sample but, differently from SEM, in this case the electrons pass through the sample and are collected thereafter. These transmitted electrons are directed to a fluorescent screen, where they will form an image giving more details about the internal structure of the membrane [89]. TEM can be used to investigate the morphology of cells and their organelles, which might be of use in diagnostic pathology studies [90].

#### 2.4.3. Small Angle and Wide Angle X-Ray Scattering

Small angle X-ray scattering (SAXS) is a non-destructive technique that can be used to analyze a wide variety of biological and non-biological structures. In SAXS analyses, a collimated and monochromatic X-ray beam hits the sample, and the scattered radiation at low angles is recorded by a detector, providing information on the structure of the sample [91]. SAXS is often used to study the conformation of proteins, which can largely affect the biologic membrane properties, possibly being an indicator of pathological conditions. Baroni and colleagues [92] used SAXS to evaluate the conformation of the cystic fibrosis transmembrane conductance regulator (CFTR), a membrane-integral chloride channel, whose mutations are responsible for the onset of cystic fibrosis. In particular, the authors applied the SAXS technique on microsomal membranes extracted from NIH/3T3 cells (a mouse embryonic fibroblast cell line), transfected with wild-type CFTR and with CFTR carrying a mutation. The SAXS data enabled the evaluation of the variations of membrane electron density profile, an indicator of alterations in the CFTR folding.

A combination of SAXS and wide angle X-ray scattering (WAXS) was employed by Nunes et al. [93] to study the interactions of different nonsteroidal anti-inflammatory drugs (NSAIDs) with the phospholipids. Indeed, the mechanism underlying the widely known gastrointestinal toxicity of NSAIDs has not been completely elucidated; among the hypotheses, it has been proposed that the NSAIDs compromise the integrity of the gastric mucosa by chemical association with the cell membrane phospholipids [94]. To simulate gut cell membranes, the authors used 1,2-dipalmitoyl-*sn*-glycero-3-phosphocholine (DPPC) liposomes, since phosphatidylcholines are the main lipid constituents of the gastric mucous layer. Mixtures composed of different amounts of NSAID and DPPC in organic solvent were dried, and liposomes were obtained by thin film hydration followed by several cycles of heating (at 60 °C) and centrifugation. SAXS and WAXS analyses on the obtained dispersions revealed that the correlation length between the bilayers was reduced in the presence of all NSAIDs tested, highlighting the disturbing effect of these drugs in membrane order.

## 3. Extracellular Vesicles: Membrane Characteristics

EVs are naturally secreted by almost all cell types, and include different vesicles usually classified on the basis of their size, cellular compartment of origin and localization [95,96]. EVs play a crucial role in cell-to-cell communications, under both physiological and pathological conditions; indeed, it is well known that EVs can shuttle bioactive molecules among healthy cells, but they are also involved in different tumor processes, such as promotion of tumor progression, angiogenesis and metastasis formation [97,98]. Among the various types of EVs, exosomes are probably the most investigated by researchers, because they are characterized by higher homogeneity (in terms of size, density, and membrane composition) compared to other EVs classes.

Exosome membrane composition is influenced by the cell of origin, but some general considerations can be made. Exosomes are constituted, to a large extent, of membrane lipids, while only minor amounts of other lipids are captured from the cytosol during the process of exosome formation and release [99].

Regarding the lipid distribution between the outer and inner leaflet of exosome membrane, opposing pieces of evidence have been reported. Indeed, some authors state that, differently from the plasma membrane, characterized by an asymmetric distribution of lipids, exosomes lack transmembrane phospholipid asymmetry; for example, Laulagnier et al. [100], studying the membranes of exosomes deriving from a leukemia cell line, reported that 70% of PE was present on exosome outer leaflet, which is compatible with a vesicle in which the asymmetrical distribution of phospholipids was lost. Also Record and colleagues [101] demonstrated this lack of asymmetry by evidencing the presence of 50–70% of aminophospholipids (PE and PS) in the outer leaflet of exosomes. According to these authors, this might be due to the absence of the enzymes flippase and floppase in the exosome membrane [102], and also to the lack of an organized cytoskeleton, contributing to the maintenance of the asymmetric composition of the plasma membrane. Conversely, other works in the literature claim the asymmetric organization of lipids even in the exosome membranes [99,103,104]. Interestingly, in a study from Yasuda and colleagues [105], the asymmetric distribution of lipids in the two leaflets of the membranes of exosomes of different origin was demonstrated by fluorescence spectroscopy; in particular, they observed that, except for milk exosomes, the asymmetry of the inner and outer leaflets of exosomes from human prostate cancer, lung cancer and embryonic kidney cells was retained. It has been hypothesized that the maintenance of the asymmetric lipid distribution in exosome membranes might be due to thermodynamic lipid–lipid and lipid–protein interactions, since the lipid flip-flops might be decreased by lateral interactions under a less fluidic membrane environment [106].

Although no consensus on the lipid composition of exosomes of different origins has been reached among researchers, there appeared to be a lot of trends conserved across various cell types [100,107,108]. Most lipids in the exosomal membrane are chol and phospholipids [109], but the exosomal membrane contains a much higher amount of chol (about 43%) than the usual organelle membrane (8–40%) [66], resulting in lower membrane fluidity [110]. Chol enrichment in endosomal membranes seems to be crucial to provide adequate conditions for exosome formation [111] and possible roles of chol in exosomes have been discussed in the literature. Chapuy-Regaud et al. [112] observed that the chol enrichment in exosomes produced by Hepatitis E virus (HEV)-infected liver cancer cells could be critical for HEV entry; indeed, it seems that the abundance of chol in these exosomes, vehiculating HEV RNA, protects them from immune response, leading to the wide circulation of HEV in its host. Furthermore, it has been hypothesized that the enrichment of chol in the exosomal membrane, increasing the mechanical strength of these vesicles, might be important in protecting their inner content during their long journey, such as cell-to-cell communication and circulation in the blood stream [113,114]. Moreover, it has been observed that, in some cases, exosome uptake by cells is mediated by chol-dependent pathways [115]; Wang et al. demonstrated that the high chol content in exosomes favors their binding to CD8 (+) T-cells [116].

Also, SM, glycosphingolipids and PS are two to three times more abundant in the exosomes than in the corresponding cells of origin, while PC decreases from 55% to approximately 30% [103,117]; finally, PE levels decrease or are maintained, depending on the parent cell type, while ceramide levels increase or remain unvaried [118].

Beyond these general considerations, it is important to underline that the lipid composition might be consistently different among exosomes of different origin; also, differences among vesicles originating from the same cell type have been reported [118]. For example, reticulocyte-derived exosomes exhibit a phospholipid composition quite similar to that of plasma membrane, with no increase in chol/phospholipid ratio, while the opposite has been reported for B cell-derived exosomes, whose chol/phospholipid ratio was increased by three times [107].

Since exosomes derive from the membrane of late endosomes, their proteome is particularly rich in heat shock proteins (HSP70, HSP90) and tetraspanins (CD9, CD63, CD81, and CD82), and they also contain a high amount of transport proteins like tubulin, actin and actin-binding molecules [119]. In particular, some tetraspanins (like CD9, CD63, CD81 and CD82) regulate the sorting of proteins and possibly the recruitment of RNA in exosomes and other EVs [120,121]. Moreover, tetraspanin proteins are involved in the exosome uptake by recipient cells [122] and regulate the adhesive activity of several adhesion molecules, including integrins, also affecting signaling pathways [123,124]. In addition, exosome membranes usually include transmembrane proteins that are specific to the parent cell; for example, exosomes deriving from dendritic cells, reticulocytes and T-cells are enriched in α- and β-chains of integrins, which play a crucial role in organ tropism transfer of exosomes (also including metastasis formation) through their interaction with extracellular matrix proteins [125,126]; instead, P-selectin and intercellular adhesion molecule-1 are present in the membranes of platelet-deriving exosomes [127].

## 4. Development of Exosome-Mimetic Drug Delivery Systems

Since the first observation of the exosomes “homing” abilities (i.e., the exosome ability to specifically target their parent cell/tissue of origin), these vesicles have been studied as promising drug carriers employable as targeted drug delivery tools [128,129]; however, their clinical application is hampered by several issues, like structural heterogeneity and complexity, difficulties in drug loading and in developing standard, scalable, and cost-effective GMP procedures for exosome isolation and purification [130].

**Figure 1:**
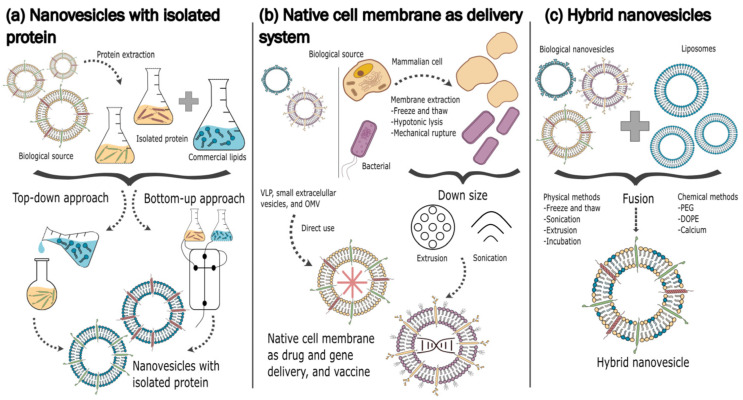
Overview of different strategies to develop biomimetic drug delivery nanosystems. (**a**) Nanovesicles with isolated proteins produced by top-down or bottom-up approaches; (**b**) biological nanovesicles, such as small extracellular vesicles and outer membrane vesicles, directly used as drug delivery systems; and (**c**) hybrid nanovesicles obtained from the fusion of biological vesicles and synthetic liposomes. Reproduced from [131] under the Creative Commons Attribution (CC BY) license (https://creativecommons.org/licenses/by/4.0/ (accessed on 28 April 2025)).

To circumvent these issues, many strategies to develop biomimetic vesicles have been proposed [131], like the coating of different kinds of nanoparticles (NPs) with isolated cell membranes [132], the development of cell membrane-derived vesicles [133], or the formulation of hybrid vesicles obtained from the fusion of EVs with synthetic vesicles [134] (Figure 1). These strategies can potentially lead to the production of vesicles under GMP conditions, for example by using GMP-compliant microfluidic systems [135]; moreover, the combination of artificial materials with biologic ones might result in cost reduction [136]; finally, by using appropriate systems for the isolation of cellular materials, a consistent decrease in batch-to-batch variability can also be assessed [137].

In recent years, great effort in developing artificial EVs has been typically focused on the surface protein profile or nucleic acids, because of their crucial role in cell interaction and communication [138,139]. Indeed, specific surface proteins can be used to achieve targeting properties; a possible “bottom–up” approach involves the incorporation of recombinant or cell-derived proteins into liposomes to create protein-functionalized liposomes [140]. In contrast, the lipidic profile generally fades into the background, recognizing in lipids only a structural role. Nonetheless, lipids are currently emerging as essential elements, with an active involvement in different biological functions [138]. Interesting studies have been reported by several research groups that optimized the development of bioinspired vesicles by studying the lipid composition and mechanical properties of the membrane of EVs and exosomes.

Moreover, when developing a nanoparticulate drug delivery system, also its mechanical properties, which may influence its interaction with cells and the cellular environment, should be optimized. Vesicle interaction with host cells might occur through different pathways, the most common being receptor-mediated endocytosis, macropinocytosis and membrane fusion [141]. The single occurring pathway and the efficacy of vesicle internalization depend upon several factors, including receptor density on the membrane of the host cell and ligand density on vesicle surface, ligand-receptor affinity, strength of attractive interactions amongst lipids in vesicles and in the cell membrane of host cells, and vesicle elasticity [141,142,143].

Yi et al. [141] conducted a theoretical study evidencing that, for a stiff particle, the adhesive interaction with the cell membrane induces the cell membrane to deform and consequently surround the particle. Conversely, in the case of soft particles, the deformation is partitioned between the particle and the membrane and, according to the authors, a very soft particle would initially spread along the cell membrane, assuming a stretched conformation, without significant deformation of the cell membrane. Therefore, since the cell membrane would be forced to bend around the stretched particle, a more abrupt rise in elastic energy would be required, while surrounding a rigid particle would involve a gentler increase in elastic energy. Consequently, to balance the faster rise in elastic energy, in the case of softer NPs, a stronger vesicle–membrane interaction is required, as also confirmed by the experimental results reported by Beningo and colleagues [144].

Also, Anselmo et al. [145] supported this hypothesis with in vivo biodistribution studies, demonstrating that softer NPs (elastic modulus = 10 kPa) exhibited longer blood circulation time compared to stiffer ones (elastic modulus = 3000 kPa), as a result of the higher uptake of stiffer NPs by macrophages of the reticuloendothelial system. In vitro internalization studies on epithelial, endothelial and macrophage cell cultures confirmed this hypothesis.

Interestingly, opposing findings were reported by other research groups: Banquy and colleagues [143] observed that macrophages preferably ingest hydrogel NPs of medium stiffness (35–130 kPa), in a larger amount and at a faster rate than soft or stiff NPs, while the study of Liu et al. [146] shows that hydrogel NPs exhibiting lower Young’s modulus were internalized at a faster rate and in a more considerable amount into hepatocyte carcinoma cells. The discrepancy between the results might be attributed to the different particle size and the different cell models; indeed, it is well known that cells differ for the capability and specific pathways to internalize exogenous particles, because of their adaptation for specific functions; moreover, the size of the particles, influencing the preferred uptake pathway, results in different uptake rate and amount.

A particular case is represented by elastic ligand-functionalized vesicles. Interestingly, when a strong ligand–receptor interaction is implicated, the mechanism described for receptor-mediated endocytic pathway changes, as demonstrated by both a molecular dynamic simulation [142] and experimental data [147]: in this specific case, to maximize the ligand–receptor interaction, the elastic vesicles deform, acquiring an oblate shape, and successively reassume their spherical shape, bypassing the high energy barrier of membrane bending to wrap the oblate vesicle.

Considering all the issues discussed in the literature, a measured stiffness–elasticity balance would be required for an effective drug delivery system: for targeted vesicles a strong ligand–receptor interaction, as well as good elasticity, are necessary for efficient endocytosis, with higher stiffness being related to unspecific uptake and lower blood circulation time. Moreover, one general way to maintain high fluidity of membranes is the incorporation of phospholipids with shorter fatty acid chains that exhibit a lower transition temperature from the gel to the liquid-crystalline state. Thus, the membrane becomes more disordered and its bilayer movements uncorrelated (e.g., dioctanoylPC (C8:0) forms micelles while dinonaoylPC (C9:0) forms unilamellar vesicles) [148].

In this context, several authors developed bioinspired liposomes, which are artificial liposomes whose lipid composition has been rationally designed based on the idea of mimicking the lipid membrane composition of exosomes [149,150]. These artificial systems have been used as models to study the effects of lipid composition on membrane properties, to investigate the cell–EVs interactions, or to develop biomimetic drug delivery systems. Interesting insights in the exosomal membrane characteristics are provided by the work of Suga et al. [114], who investigated the composition of bovine milk-derived exosomes by extracting the lipids with a methanol/chloroform mixture, and analyzed them by thin layer chromatography. This analysis suggested that milk exosomes are composed of zwitterionic lipids (SM and PC) and anionic lipids (PS and cardiolipin). Based on these experimental data, different liposomal formulations containing chol, milk SM, 1-palmitoyl-2-oleoyl-*sn*-glycero-3-PC, and cardiolipin were prepared using the thin film hydration method followed by freeze-thawing and extrusion. Exosome and liposome membranes were investigated for their fluidity and polarity by using fluorescent dyes. The membrane property of exosomes was similar to the one of liposomes composed of chol/milk SM/1-palmitoyl-2-oleoyl-*sn*-glycero-3-PC at 40/15/45 molar ratio. In particular, the authors observed that the most important features to reproduce the exosome membrane properties were the chol content, that should be around 40 mol% (since the chol content in milk exosomes was 43 mol%), and, to a lesser extent, the chol/SM ratio.

Lopez et al. [149] developed bioinspired liposomes mimicking the physicochemical properties of cancer-derived EVs as a model to study EVs properties and their impact on cellular uptake. They compared size and zeta potential of EVs isolated from different cancer cells and human fibroblast cells with bioinspired liposomes. Using microfluidics, they developed two liposome formulations: anionic liposomes, composed of 1,2-dimyristoyl-*sn*-glycero-3-PC (50 mol%), chol (40–50 mol%) and dihexadecyl phosphate (DHP, 0–10 mol%), with DHP % progressively increasing replacing chol; and cationic liposomes composed of 1,2-distearoyl-*sn*-glycero-3-PC (DSPC, 25 mol%), 1,2-dioleoyl-*sn*-glycero-3-PE (DOPE, 12.5 mol%), chol (37.5–57.5 mol%) and 1,2-dioleoyl-3-trimethylammonium-propane (DOTAP, 5–25 mol%), with DOTAP % progressively increasing replacing chol. Noteworthy, the authors used a central circumscribed composite design to optimize the liposomal formulations by varying microfluidic parameters, like the total flow rate and the flow rate ratio, and the DHP content. Then, after verifying EV internalization on human hepatocyte cells by confocal microscopy, the authors also proved that anionic and cationic liposomes were internalized, with the latter showing a higher degree of internalization, although associated with a consistent cytotoxic effect, typical of cationic NPs. Then, the effect of liposome zeta potential and size on cellular internalization was analyzed by confocal imaging and flow cytometry, revealing that smaller particles with higher zeta potential exhibited a higher uptake by cells.

Several studies reported in the literature focus on the rational formulation of exosome-mimetic systems for drug delivery, as summarized in Table 2.

For example, Martinez-Lostao et al. [151] formulated large unilamellar vesicles (LUVs) replicating the lipid composition of natural exosomes, to treat rheumatoid arthritis; the authors did not specify the origin of the reference exosomes, but referred to some unpublished data. They prepared liposomes by thin film hydration followed by freeze-thawing and extrusion, composed of PC, SM, and ovine wool chol in a 55:30:10 weight ratio, adding different amounts of 1,2-dioleoyl-*sn*-glycero-3-{[*N*-(5-amino-1-carboxypentyl)-iminodiacetic acid]succinyl}(nickel salt), exploited to conjugate to the LUV surface APO2L/TRAIL-His10 (a TNF superfamily member, potentially useful in the treatment of autoimmune diseases). The authors demonstrated that more than 90% of LUVs were conjugated with the APO2L/TRAIL-His10 by exploiting both sodium dodecyl sulfate-gel electrophoresis and a flow cytometry assay employing a fluorescent primary mouse anti-human APO2L antibody. In vivo tests on a rabbit model of antigen-induced arthritis showed 62% improvement in disease symptoms in the group treated with the APO2L/TRAIL-His10-loaded LUVs, resulting more effective if compared to the group receiving free APO2L/TRAIL-His10, exhibiting only 30% improvement. Moreover, histological analysis revealed that the treatment with APO2L/TRAIL-His10-loaded LUVs led to greater reduction in synovial hyperplasia and inflammatory infiltrates compared to soluble APO2L/TRAIL-His10. The authors also assessed the absence of systemic toxicity of the developed nanosystem, and concluded that their exosome-mimicking liposomes provide a promising therapeutic strategy for rheumatoid arthritis treatment; however, data regarding the exosome-mimicking liposome characterization (like size, zeta potential, mechanical properties) are lacking, as well as a clear reference for the lipid composition choice.

To target dendritic cells, Li and his group [152] prepared an exosome-mimetic nanosystem functionalized with an antibody against DEC205 (one of the major endocytic receptors on dendritic cells [158]), by using a novel self-emulsifying method. First, a microemulsion composed of an oil phase of PC and Cremophor EL in different weight ratios from 1:9 to 9:1, and an aqueous phase composed of a bovine serum albumin solution, was prepared by the water titration method. Then, this microemulsion was mixed with a suspension of micelles composed of DOPE and 3-(*N*-(*N*′,*N*′-dimethylaminoethane)carbamoyl)chol (DC-chol) and, after evaporation of the organic solvent and sonication, they obtained nanosized liposomes composed of an inner microemulsion surrounded by a DOPE and DC-chol shell. Interestingly, the authors optimized the formulation by using a Box-Behnken design. To conjugate the anti-DEC205 antibody to the liposome surface, the outer shell also included cholesteryl succinate (CHS), in DOPE/DC-chol/CHS 8:2:1 weight ratio; therefore, DEC205-functionalized liposomes were obtained by EDC coupling of the amino groups of the antibody with the activated ester bonds. The conjugation efficiency, calculated by purifying the liposomes by gel filtration chromatography and measuring the unconjugated antibody by Bradford assay, was 61 ± 4%. Untargeted and targeted liposomes exhibited a mean diameter of 63 ± 6 nm and 82 ± 4 nm, respectively, with positive zeta potential values (+31 ± 2 mV and +20 ± 2 mV). The encapsulation efficiency (EE%) was calculated by quantifying the unencapsulated albumin, separated by gel filtration chromatography, by Bradford assay; after investigating the effect of formulation ingredients on EE%, the authors obtained mean EE% of 91 ± 3% and 93 ± 2% for untargeted and targeted liposomes, respectively. TEM images assessed the round-shaped morphology of the liposomes. Finally, the authors proved reduced cytotoxicity, tested in vitro on dendritic cells by MTT assay, of the targeted compared to the untargeted liposomes, probably because the antibody coupling resulted in a shedding of the positive charge of the vesicles, known to be cause of toxic effects; moreover, flow cytometry analysis showed higher internalization of the targeted nanoparticles on dendritic cells, with respect to the untargeted ones. The authors conclude that their novel exosome-biomimetic nanosystem might represent a promising carrier for targeting therapeutics to dendritic cells, but they do not highlight how this system effectively mimics naturally secreted exosomes, and they refer to neither literature works nor preliminary data to justify the choice of the lipid composition of the system.

The group of Lu et al. [153] developed liposomes with lipid composition, size, and zeta potential mimicking exosomes for delivery of a VEGF-targeting siRNA (siVEGF). Also in this case, the authors did not focus on the lipid composition of exosomes deriving from a specific type of cells, but based their formulation on studies reporting general trends in the exosome membrane lipid composition. They prepared liposomes composed of DOPC, SM, chol, 1,2-dioleoyl-*sn*-glycero-3-PS (DOPS), and DOPE at a 21:17.5:30:14:17.5 mol%, while DOTAP liposomes (DOTAP/DOPC/chol at a 40:40:20 mol%) and PC liposomes (DOPC/chol at a 70:30 mol%) were prepared as comparison; all formulations were prepared by hydration of the lipid film with a siRNA aqueous solution, followed by extrusion. The mean size (119 nm) and zeta potential (−24 mV) of the exosome-mimicking formulation were comparable to those of exosomes. The siVEGF EE% was investigated by using a fluorescently labelled siRNA; in particular, the liposomes loaded with the fluorescent siRNA were purified by ultrafiltration, and the EE% was calculated on the basis of unencapsulated siRNA. This method might be affected by a high error, because it does not take into account that the siRNA that is not found in the unencapsulated fraction might be aspecifically adsorbed on the filter or might simply get lost during the preparation procedures. As expected, the highest siRNA EE% values were found for DOTAP liposomes; however, it has to be considered that cationic liposomes present some cytotoxicity issues [159]. After 90 day-storage at 4–8 °C, the exosome-mimicking liposomes resulted more stable than DOTAP and PC liposomes, probably because of the elevated rigidity of the SM/chol bilayer membrane [160]. Then, the authors investigated liposome internalization on A549 (lung adenocarcinoma) and HUVEC (human umbilical vein endothelial cells), observing that the DOTAP liposomes, thanks to their positive charge, showed the highest degree of internalization, followed by the exosome-mimicking liposomes, whose internalization was significantly higher than that of PC liposomes in both cell lines. Interestingly, the exosome-mimicking liposomes exhibited a higher negative surface charge than the PC liposomes, and this implies that the unique lipid composition of exosome-mimicking liposomes may contribute to the enhanced cellular internalization. The authors also demonstrated that exosome-mimicking liposome internalization was dependent on caveolae-mediated endocytosis, macropinocytosis, and membrane fusion, similarly to what previously observed for exosomes. Gene silencing efficacy studies displayed results similar to the internalization studies: the maximum silencing effect was observed in cells treated with the siVEGF DOTAP-liposomes, followed by the siVEGF exosome-mimicking liposomes, exhibiting a three-fold higher effect than siVEGF PC-liposomes.

To achieve the targeted delivery of therapeutic oligonucleotides to lung adenocarcinoma cells, Vázquez-Ríos and her group [154] developed an exosome-mimetic nanosystem (EMN) simulating the structure and functionality of tumor-derived exosomes. These authors, after performing proteomic analysis on exosomes isolated by ultracentrifugation from different cancer cells, and reviewing the literature reporting lipidomic studies on exosomes, formulated their EMN composed of chol:PC:SM:C16 ceramide (0.9:1:0.4:0.03 *w*/*w*) by ethanol injection method. EMNs were loaded with miR145, recently revealing its role as a tumor suppressor in several cancers [161], by dissolving it in ethanol prior to injection into the aqueous phase. ^1^H- and ^31^P-NMR analyses proved that each lipid component was effectively incorporated into EMNs, also confirming a proportion of SM/PC equal to 0.48:1, similar to the theoretical one. Confocal images showed the effective internalization of these EMNs on different cancer cell lines. To better mimic exosomes, the authors increased the complexity of the formulation by functionalizing the EMNs with different proteins, typically present on exosomes surface, and verified that these functionalized miRNA-loaded EMNs and tumor-derived exosomes exhibited similar capacity—to transport therapeutic RNAs to cancer cells. This aspect and the time- and cost-saving production method, which can be easily transferred to GMP production, make this exosome-mimetic nanosystem very promising.

Sakai-Kato et al. [102], basing on previous studies reporting the lipid membrane composition of exosomes deriving from a human hepatocellular carcinoma cell line [99], designed an artificial liposome mimicking the lipid composition and the physicochemical characteristics of these vesicles. The exosomes were isolated by using a commercially available exosome isolation kit, while liposomes with different lipid compositions were prepared; in particular, the authors considered that the main lipid composition of the Hep-G2 cell-derived exosomes is reported to be PC (20%), chol (40%), PS (15.6%) and SM (10%). Therefore, considering that the most abundant PC species in exosomes is DSPC, while the most abundant PS are 18:0 and 18:1 lipids, the following formulations were prepared: DSPC/chol/DOPS (40:40:20 mol%), SM/DSPC/chol/DOPS (10:30:40:20 mol%), DSPC/chol/1,2-distearoyl-*sn*-glycero-3-PS (DSPS) (40:40:20 mol%), SM/DSPC/chol/DSPS (10:30:40:20 mol%), SM/DSPC/chol/1,2-dioleoyl-*sn*-glycero-3-phosphoglycerol (DOPG) 10:30:40:20 mol%), and DSPC/chol (60:40 mol%). The liposomes were obtained using the thin film hydration technique; then, they were either extruded or subjected to freeze-thaw cycles, followed by sonication and extrusion. All the liposomal formulations were monodispersed and exhibited nanometric size (between 100 and 150 nm) and negative zeta potential. By AFM, exosomes showed significantly lower stiffness compared to saturated lipid/chol liposomes, while the exosome stiffness was higher than that of liposomes composed of unsaturated lipids/chol. Interestingly, the authors observed that SM addition slightly softened the liposome, contrary to what was expected due to its long acyl chain and saturation. Conversely, the stiffness of SM/DSPC/chol/DSPS and DSPC/chol/DOPS liposomes was not significantly different compared to exosomes. Following in vitro studies using fluorescently labeled liposomes were conducted to assess the liposome internalization abilities on HeLa cells, by confocal imaging. These studies evidenced that the role of SM in the cellular internalization of liposomes was limited, and that DOPS-containing liposome internalization was twice as high as that of DSPS-containing liposomes. Finally, after demonstrating that liposome internalization was mediated by an active transport mechanism by observing a dramatic decrease of internalized liposomes when the experiment was conducted at 4 °C, the authors proved the involvement of TIM4 transmembrane protein, which is recognized by PS [162]; the intracellular fluorescence intensity, related to internalized liposomes, significantly decreased when the cells were pre-incubated with an anti-TIM4 antibody. In a following work [155], the group also employed the optimized DSPC/chol/DOPS (40:40:20 mol%) formulation to study the effect of sample concentration on nanoparticle tracking analysis.

In another study [156], a novel approach for the preparation of extracellular vesicles-mimicking lipid nanoparticles incorporating plasmid DNA, performed through micropipette mixing, syringe pump or microfluidics, was disclosed. In fact, loading nucleic acids into extracellular vesicles is challenging due to the negative charge of the EV lipid components. To overcome this issue, protamine sulfate, an arginine-rich protein, is employed to complex the nucleic acids in an aqueous solution before being mixed with lipids in an ethanol phase. Due to challenges in reproducibility and the efficiency of this method, the authors conceived the dispersal of all components, namely protamine sulfate, plasmid DNA and lipids chosen on the basis of the lipidomics study performed by the afore-cited Skotland et al. [99], in an alcohol phase; the subsequent injection of the alcoholic dispersion into an aqueous solution induces the formation of lipid NPs. In this valuable research, the authors developed an alternative one-step approach: after testing various alcoholic compositions, they chose to disperse plasmid DNA and protamine sulfate in propylene glycol containing a small volume of water and sodium chloride to prevent precipitation, followed by addition of an ethanolic solution of DOPC:DOPE:DOPS:SM:chol (the most abundant lipids present in EVs) in an 18:7:13:17:45 mol% ratio, including a PEGylated lipid (*N*-octanoyl-sphingosine-1-{succinyl[methoxy(polyethyleneglycol)2000]}, and 1 mol% of total lipids) to improve dispersion. The injection of the alcohol phase into a pH 4.4 acetate buffer followed by further dilution with PBS let obtain the lipid NPs, purified by ultrafiltration. Among the three different preparation methods, microfluidics was the preferred one, thanks to its reduced variability in terms of particle size, gene expression efficiency and high reproducibility. In Hep-G2 cells the lipid NPs, despite having negative zeta potential and being devoid of surface antigens, exhibited transfection efficiencies comparable to those of cationic lipoplexes. The mechanism of NP internalization was studied, and the results suggested a combination of lipid raft-mediated endocytosis and macropinocytosis. The authors hypothesize that one or more lipids in their particles (and this might apply also to EVs) might interact with some serum proteins, inducing the formation of a protein corona; on the other hand, macropinocytosis might occur constitutively in Hep-G2 cells due to their KRAS expression.

**Figure 2:**
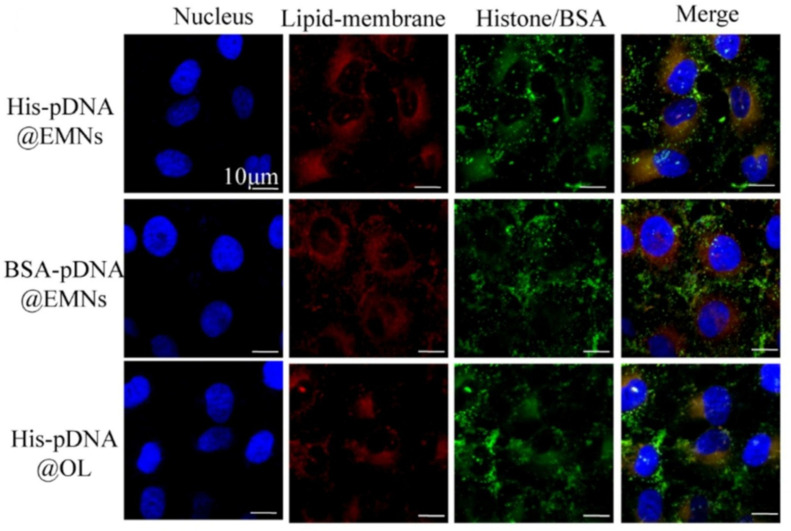
Co-localization analysis of Pigment Epithelium-Derived Factor with histone or bovine serum albumin in HUVEC for His-pDNA@EMNs, BSA-pDNA@EMNs, and His-pDNA@OL (His-pDNA@ordinary liposomes; red: Cy5.5-labeled PEDF-pDNA; green: FITC-labeled Histone or BSA; blue: nucleus). Reproduced with permission from [26].

To replicate the gene transfer properties of exosomes, while overcoming the issues hampering the clinical translation of such vesicles, Li et al. [26] developed a liposomal formulation, composed of SM, PC, PS, PE and chol and loaded it with Pigment Epithelium-Derived Factor genes linked with a histone (His-pDNA) aimed at gene transfection. The liposomes were prepared by using the double emulsion method, with the oil phase composed by a mixture of chol, SM, PC, PS, PE in a 10:10:15:10:35 mol% ratio in dichlorometane; the lipid composition was based on the lipidomics analysis of HUVEC-derived exosomes, avoiding the use of glycolipids because, in spite of being the most abundant, they possess a non-uniform lipid structure. Then, the internal aqueous phase including the active was combined with the oil phase, creating the primary emulsion that was then mixed with an external aqueous phase to form the final emulsion. Finally, nitrogen gas was passed through the water to remove the dichloromethane, and obtain the exosome-mimetic NPs. To specifically study the role of the singular lipid components, formulations lacking SM, PC, PS or PE were also assembled. The results of the study showed that HUVEC exhibit rapid uptake of these NPs (within the initial 2 h the uptake was 92% higher than ordinary liposomes obtained with chol/lecithin 34.5:65.5 weight ratio); the mechanism for internalization was found to be clathrin-mediated endocytosis (Figure 2). When analyzing the internalization results of vesicles stripped of the individual components, PC favored the uptake, while PE and PS increased the lysosomal escape rate, and PS and SM showed the importance of their role in nuclear entry. All these results complied with the study of transfection ability. Finally, the authors studied the relationship of phase transition temperature with the lysosome escape efficiency by differential scanning calorimetry measurements. The NPs with complete lipid composition exhibited the lowest phase transition temperature, while the formulations devoid of SM, PC, PE and PS showed gradually increasing phase transition temperatures, respectively. These results confirm the relationship between the efficiency of lysosomal escape and the rigidity and physical structure of the vesicle membranes. Specifically, vesicles whose membrane features lower phase transition temperature are less rigid, which facilitates the fusion between the vesicle membrane and the lysosomal membrane.

**Figure 3:**
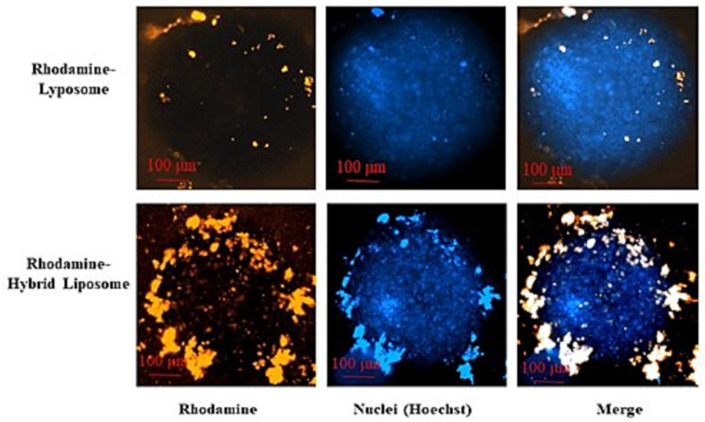
Cell uptake of fluorescent hybrid liposomes in 3D melanoma model. The upper panel shows the fluorescence intensities of rhodamine labelled-liposomes (orange), Hoechst (blue, nuclei), and the merge of them; the bottom panel shows the fluorescence intensities of rhodamine labelled-hybrid liposomes (orange), Hoechst (blue, nuclei), and merge of them. Scale bar 100 μm. Reproduced from [157] under the Creative Commons Attribution (CC BY) license (https://creativecommons.org/licenses/by/4.0/ (accessed on 28 April 2025)).

Differently from the works reported previously in this section, Arduino et al. [157] developed hybrid liposomes, obtained from the fusion of cell membranes isolated from a metastatic melanoma cell line and synthetic lipids. In particular, L-α-PC (L-α-phosphatidylcholine) and chol were selected as synthetic lipids because, even if these lipids do not enhance the interaction with cells, they exhibit structural and functional role in mimicking biological membranes, besides being biocompatible. Moreover, the authors state that the PC/chol ratio, set at 70:30 mol%, was based on both preliminary experiments and literature observations, reporting that this ratio is the most suitable one to obtain a conveniently flexible formulation, able to release drugs with different physicochemical characteristics [163,164]. To isolate the cell membrane fraction, the cell pellet was lysed by addition of milliQ water, then it was subjected to freeze-thawing, sonication and ultracentrifugation. The protein content of the resuspended membranes was measured by Bradford assay. The hybrid liposomes were prepared by using two different 3D-printed microfluidics devices; noteworthy, since the authors presume that the hydrodynamic forces in the microchannels might not be sufficient to disrupt cell membranes, necessary for their mixing with synthetic lipids and formation of hybrids, during the preparation of the formulations the microfluidic devices were immersed in an ultrasonic bath, thus adding an active mixing component (due to the external physical forces) to the passive mixing induced by the geometry of the microfluidic chip. The organic phase was composed of PC and chol, with the addition of cobimetinib or lenvatinib (actives employed in the treatment of melanoma), while the aqueous phase comprehended the suspension of the isolated cell membranes in phosphate buffered saline; different formulations were prepared by modulating the synthetic lipids/cell membranes (quantified as protein content) ratio. The obtained vesicles, whose effective hybridization was verified by Förster resonance energy transfer analysis, were nanosized and negatively charged, with an EE%, measured spectrophotometrically, over 75%. Finally, the authors demonstrated the higher and specific internalization of the drug-loaded hybrid liposomes on the parent melanoma cells using both 2D cell cultures and 3D spheroids (Figure 3), compared to the corresponding synthetic liposomes. However, a comparison with the pure cell membrane-derived vesicles might be of interest, to evaluate the effective convenience in the fabrication of a hybrid system.

## 5. Conclusions

This review provides an overview on the complex structure and heterogeneity of the membrane bilayer, which consists of different types of lipids and proteins, and highlights how this structure is crucial in determining both the integrity and functional dynamics of cells. The review emphasizes how certain physico-chemical characteristics of lipids, such as chain length, saturation, and head group size, have a significant impact on membrane fluidity, thickness and curvature, and how these characteristics, in turn, modulate important biological processes like signal transduction, cellular trafficking, and intercellular communication. In this review, also the crucial role of membrane proteins in preserving the structural and functional blueprint of cell membranes is underlined. Cell membranes display a complex interplay of lipid–protein interactions, and even minor changes in the structure and composition of lipids can have a major impact on membrane rigidity and permeability, which are ultimately connected to pathological conditions like cancer and other diseases.

The review also highlights the importance of characterizing both cell and EV membrane in the rational design of bioinspired therapeutic systems.

In addition, progress towards characterizing membrane biomechanics is herein described. The integration of lipidomics and proteomics analyses with biophysical measurements, using tools such as AFM, allowing to probe changes in stiffness and elasticity in diseased cells, provide a better understanding of membrane behavior that is essential for fundamental cell biology, as well as for disease diagnosis and development of drug delivery systems.

Finally, this review reports examples of innovative drug delivery systems that have been rationally formulated with the aim of reproducing the lipid composition and/or the mechanical properties of biological membranes.

New biophysical knowledge of natural cell membrane features, combined with biomimetic design, might drive the rational design of novel platforms for drug delivery, possibly enhancing the therapeutic outcomes. More specifically, improving our knowledge of the cell membrane structure and features, especially when applied to patient-specific cells, can help researchers to develop diagnostic and therapeutic tools that can better reflect the properties shown by the cell membrane when affected by diseases, leading the way in the precision medicine field.

### Future Directions

Deepening the knowledge in the composition and mechanical properties of biological membranes might lead to the rational formulation of nanosystems that mimic endogenous structures in a more accurate way, possibly resulting in enhanced targeting efficiency, increased biocompatibility and reduced risk of immune response and rapid clearance.

Therefore, the application of techniques such as AFM, SAXS and microscopy to the characterization of biological membranes, as well as bioinspired drug delivery systems, should become more diffuse.

Finally, the development of biomimetic drug delivery systems based on the above-mentioned approach should always be accompanied by objective evaluations of their industrial scalability.

## Figures and Tables

**Table 1:** Main lipid components of cell membranes and relative distribution and properties.

Lipid	Ratio (mol%)	Membrane Leaflet	Mechanism Maintaining the Asymmetric Distribution	Properties
Phosphatidic acid	1	Inner leaflet	-	Levels regulated by diacylglycerol kinase and phospholipase D, elevated levels activate kinases involved in cancer cell metabolism and proliferation.
Phosphatidylinositol	5–12	Inner leaflet	-	Substrate for phospholipase C or phosphoinositide-3 kinase, regulating role in membrane trafficking and proliferation.
Phosphatidylglycerol	1	Inner leaflet	-	High level observed in increased viral replication and cancer development.
Phosphatidylserine	6	Inner-outer leaflet	Calcium ion-dependent flippase moves PS to the outer leaflet; exposed on the outer leaflet during cellular oxidative stress	Role in apoptosis and protecting cells from immune detection; in some cases, it can be considered a tumor marker.
Phosphatidylethanolamine	15–25	Inner leaflet	Change from lamellar to hexagonal II phase to enhance the fusion of lipid bilayers with lysosomal membranes, reverse distribution in cancer cells	Lipid chaperone that assists membrane protein folding, involved in cell signaling pathways; promotes autophagy and regulates protein interactions.
Phosphatidylcholine	40–50	Outer leaflet	-	Choline kinase alpha and phospholipases C and D regulate its metabolism; its level can affect cell proliferation and energy metabolism in cancer cells.
Sphingomyelin	10–20	Outer leaflet	-	Involved in molecular sorting, cell-cell interaction, intracellular transport and signaling.
Cholesterol	20–50	Depends on the affinity with other components	Stronger interaction with sphingolipids: outer leaflet/major affinity with PE and PS: inner leaflet (60%)	Regulates membrane permeability, affects stiffness, thickness and thermosensitivity.

**Table 2:** Rationally formulated biomimetic drug delivery systems.

Ref.	Type of System	Composition	Inspiring Vesicles	Application	In Vitro/In Vivo Tests
[114]	Liposomes	(1) Chol/milk SM/POPC 40:15:45 mol%, (2) Chol/milk SM/POPC/cardiolipin 40:15:30:15 mol%	Bovine milk-derived exosomes	Drug delivery of nucleic acid	
[151]	Large unilamellar vesicles	PC/SM/ovine wool chol in 55:30:10 weight ratio + different amounts of 1,2-dioleoyl-*sn*-glycero-3-{[*N*-(5-amino-1-carboxypentyl)-iminodiacetic acid]succinyl}(nickel salt)	Exosomes of unspecified origin	Treatment of autoimmune diseases	In vivo tests on a rabbit model of antigen-induced arthritis
[152]	Liposomes	PC/Cremophor EL in different weight ratios from 1:9 to 9:1 + bovine serum albumin + DOPE/DC-chol/cholesteryl-succinate in 8:2:1 weight ratio	Exosomes of unspecified origin	Dendritic cell targeting	In vitro: cytotoxicity (by MTT) and internalization (flow cytometry) on dendritic cells
[153]	Liposomes	(1) DOPC/SM/chol/DOPS/DOPE in 21:17.5:30:14:17.5 mol%, (2) DOTAP/DOPC/chol in 40:40:20 mol%, (3) DOPC/chol in 70:30 mol%	Exosomes of unspecified origin	Delivery of a VEGF-targeting siRNA	In vitro: cytotoxicity (by MTT), internalization on A549 (lung adenocarcinoma) and HUVEC cells (confocal laser scanning microscopy), uptake efficiency (flow cytometry and fluorescence microscopy) and gene silencing efficacy studies
[154]	Exosome-mimetic nanosystem	Chol/PC/SM/C16 ceramide in 0.9:1:0.4:0.03 weight ratio + protein functionalization	Tumor-derived exosomes	Delivery of miR145, as an anticancer active	In vitro: internalization on SW480 (colorectal cancer), PC-3 (prostatic adenocarcinoma) and A549 (lung adenocarcinoma) cells by confocal microscopy
[102,155]	Liposomes	(1) DSPC/chol/DOPS in 40:40:20 mol%, (2) SM/DSPC/chol/DOPS in 10:30:40:20 mol%, (3) DSPC/chol/DSPS in 40:40:20 mol%, (4) SM/DSPC/chol/DSPS in 10:30:40:20 mol%, (5) SM/DSPC/chol/DOPG in 10:30:40:20 mol%, (6) DSPC/chol in 60:40 mol%	Exosomes deriving from human hepatocellular HepG2 carcinoma cells	Targeted drug delivery and intracellular trafficking studies	In vitro: internalization on HeLa cells by confocal microscopy
[156]	EV-mimicking lipid nanoparticles	DOPC/DOPE/DOPS/SM/chol in 18:7:13:17:45 mol% + *N*-octanoyl-sphingosine-1-{succinyl[methoxy(polyethyleneglycol)2000]}, 1 mol% of total lipids	Lipidomic study on extracellular vesicles	Delivery of plasmid DNA for transfection	In vitro: internalization on Hep-G2 cells by measuring the transfection efficiency
[26]	Liposomes	Chol/SM/PC/PS/PE in 10:10:15:10:35 molar ratio and formulations lacking SM, PC, PS or PE	Lipidomics analysis of HUVEC-derived exosomes	Delivery of Pigment Epithelium-Derived Factor genes linked with a histone (aimed at gene transfection)	In vitro: Internalization on HUVEC by flow cytometry; in vivo: tests on a mouse model of High Altitude Pulmonary Edema
[157]	Hybrid liposomes	Cell membranes isolated from BRAF wild type metastatic melanoma cell line, L-α-PC and cholesterol in 70:30 mol%	/	Delivery of cobimetinib or lenvatinib for melanoma treatment	In vitro: internalization on the parent melanoma cells by flow cytometry and confocal microscopy (on 2D cell cultures and 3D spheroids), hemolysis test on whole human blood, cytotoxicity on melanoma cells (by MTT)

## Abbreviations

The following abbreviations are used in this manuscript:

α-syn	α-synuclein
AFM	atomic force microscopy
BSE	back-scatter electron
CFTR	cystic fibrosis transmembrane conductance regulator
chol	cholesterol
CHS	cholesteryl succinate
DC-chol	3-(*N*-(*N*′,*N*’-dimethylaminoethane)carbamoyl)cholesterol
DGK	diacylglycerol kinase
DHP	dihexadecyl phosphate
DLPC	1,2-dilauroyl-*sn*-glycero-3-phosphatidylcholine
DOPC	1,2-dioleoyl-*sn*-glycero-3-phosphatidylcholine
DOPE	1,2-dioleoyl-*sn*-glycero-3-phosphatidylethanolamine
DOPG	1,2-dioleoyl-*sn*-glycero-3-phosphoglycerol
DOPS	1,2-dioleoyl-sn-glycero-3-phosphatidylserine
DOTAP	1,2-dioleoyl-3-trimethylammonium-propane
DPPC	1,2-dipalmitoyl-*sn*-glycero-3-phosphocholine
DSPC	1,2-distearoyl-*sn*-glycero-3-phosphatidylcholine
DSPS	1,2-distearoyl-*sn*-glycero-3-phosphatidylserine
EE%	encapsulation efficiency
EMN	exosome-mimetic nanosystem
EV	extracellular vesicle
Fb	breakthrough force
GPMV	giant plasma membrane vesicle
HEV	Hepatitis E virus
His-pDNA	Pigment Epithelium-Derived Factor genes linked with a histone
LUV	large unilamellar vesicle
NP	nanoparticle
NSAID	nonsteroidal anti-inflammatory drug
PC	phosphatidylcholine
PE	phosphatidylethanolamine
PG	phosphatidylglycerol
PI	phosphatidylinositol
PS	phosphatidylserine
PSPD	position-sensitive photo diode
SAXS	small angle X-ray scattering
SE	secondary electron
SEM	scanning electron microscopy
siVEGF	vascular endothelial growth factor-targeting siRNA
SM	sphingomyelin
TEM	transmission electron microscopy
VEGF	vascular endothelial growth factor
WAXS	wide angle X-ray scattering
